# Treatment of Tonsillitis

**Published:** 1884-01

**Authors:** 


### Treatment of Tonsillitis.

Dr. S. Solis Cohen gives the following treatment, which he says is pursued at the Philadelphia Polyclinic with eminent success:

1. In simple inflammatory tonsillitis, take two fluid drachms each of the ammon. tinct. of guiac. and the comp. tinct. of cinchona, mix with six fluid drachms of clarified honey and shake together until the sides of the vessel are well coated; add gradually a solution of eighty grains of chlorate of potassium in four ounces of water, shaking meanwhile. This is to be used as a gargle every one-half to three hours. Relief is usually experienced within a few hours and recovery is prompt. A saline cathartic may accompany the use of the gargle. None of the cases seen suppurated, and if seen within the first twenty-four hours such accidents are very unlikely.

2. In rheumatic or constitutional tonsillitis (characterized by intense pain in swallowing, causing great accumulation of saliva from unwillingness to swallow, with slight, perhaps no, congestion of throat, and subsequent fever; one or both tonsils becoming enlarged after some hours as the febrile symptoms decline, and muscular or joint rheumatism sometimes developing later), after a saline cathartic, give the following in tablespoonful doses every two hours:

| | | |
|---|---|---|
| ℞ | Sodii salicylate, | ʒ ij, |
| | Ol. gaultheriæ, | ♏ j, |
| | Liq. ammon. citrat., | |
| | Syrup simp., | ℥ ij. |

Lengthening the intervals as the pain subsides. Pieces of ice or guiac gargle promote comfort and the stiff neck is best relieved by faradization. Salicylate of quinine or cinchonidine may be substituted for the above if a tonic be required, in five-grain doses every four to six hours.—*Medical News.*

